# Human Adipose Derived Cells in Two- and Three-Dimensional Cultures: Functional Validation of an In Vitro Fat Construct

**DOI:** 10.1155/2020/4242130

**Published:** 2020-06-10

**Authors:** Robert Bender, Michelle McCarthy, Theodore Brown, Joanna Bukowska, Stanley Smith, Rosalyn D. Abbott, David L. Kaplan, Christopher Williams, James W. Wade, Andrea Alarcon, Xiying Wu, Frank Lau, Jeffrey M. Gimble, Trivia Frazier

**Affiliations:** ^1^LaCell LLC, New Orleans LA, USA; ^2^Center for Stem Cell Research and Regenerative Medicine, Tulane University School of Medicine, New Orleans LA, USA; ^3^Polish Academy of Science, Olsztyn, Poland; ^4^Department of Biomedical Engineering, Carnegie Mellon University, Pittsburgh PA, USA; ^5^Departments of Biomedical Engineering and Chemical and Biological Engineering, Bioengineering and Biotechnology Center, Tufts University, Medford MA, USA; ^6^Department of Pharmacology, Xavier University of Louisiana, New Orleans LA, USA; ^7^Plastic and Reconstructive Surgery, Baton Rouge LA, USA; ^8^Obatala Sciences Inc., New Orleans LA, USA; ^9^LSU Department of Plastic Surgery, New Orleans, LA, USA; ^10^Department of Structural and Cell Biology, Tulane University School of Medicine, New Orleans LA, USA; ^11^Department of Medicine, Tulane University School of Medicine, New Orleans LA, USA; ^12^Department of Surgery, Tulane University School of Medicine, New Orleans LA, USA

## Abstract

Obesity, defined as a body mass index of 30 kg/m^2^ or above, has increased considerably in incidence and frequency within the United States and globally. Associated comorbidities including cardiovascular disease, type 2 diabetes mellitus, metabolic syndrome, and nonalcoholic fatty liver disease have led to a focus on the mechanisms promoting the prevention and treatment of obesity. Commonly utilized *in vitro* models employ human or mouse preadipocyte cell lines in a 2-dimensional (2D) format. Due to the structural, biochemical, and biological limitations of these models, increased attention has been placed on “organ on a chip” technologies for a 3-dimensional (3D) culture. Herein, we describe a method employing cryopreserved primary human stromal vascular fraction (SVF) cells and a human blood product-derived biological scaffold to create a 3D adipose depot in vitro. The “fat-on-chip” 3D cultures have been validated relative to 2D cultures based on proliferation, flow cytometry, adipogenic differentiation, confocal microscopy/immunofluorescence, and functional assays (adipokine secretion, glucose uptake, and lipolysis). Thus, the *in vitro* culture system demonstrates the critical characteristics required for a humanized 3D white adipose tissue (WAT) model.

## 1. Introduction

Obesity is defined as a body mass index (BMI), calculated as (weight (kg)/height^2^ (m), of 30 or above [1]. This condition can be the consequence of both hyperplasia and hypertrophy of mature adipocytes and their progenitor stromal/stem cells within adipose tissue depots [1]. According to the Centers for Disease Control, the past three decades have witnessed a considerable increase in the incidence and frequency of obesity in the United States where levels of >35% exist in some states (http://www.cdc.gov/obesity/data). While few other countries approach this level of obesity, many are experiencing alarming increases due to changes in diet, exercise, and lifestyle. Since obesity is accompanied by comorbidities including cardiovascular disease, type 2 diabetes mellitus, metabolic syndrome, and nonalcoholic fatty liver disease, the international scientific community has focused its attention on the mechanisms promoting the prevention and treatment of obesity [1]. In vitro models employ preadipocyte cell lines, such as 3T3-L1, or primary adipose-derived stromal/stem cells (ASC) derived from rodent or human adipose tissue [2–4]. Over a one- to two-week period, these cultures undergo robust adipogenesis in response to inductive cocktails containing glucocorticoid and peroxisome proliferator-activated receptor *γ* (PPAR*γ*) ligands, a phosphodiesterase inhibitor to elevate cyclic AMP levels, and insulin. Nevertheless, a recent comprehensive scholarly review has concluded that there is a pressing need to improve preadipocyte models in order to enhance discoveries relating to adipocyte biology and dysfunction in obesity research [5].

While the majority of *in vitro* studies have employed a 2-dimensional (2D) format, this structural approach fails to mimic the native 3-dimensional (3D) microenvironment. Mature adipocytes in vivo are surrounded on all sides by a biomechanically supportive extracellular matrix (ECM) and display a classical “signet ring” morphology, characterized by a single unilocular lipid vacuole occupying the entire cytoplasmic space with an eccentric nucleus. In contrast, adipocytes in 2D cultures *in vitro* display multilocular lipid vacuoles scattered throughout the cytoplasm around a centrally located nucleus. Morphological differences appear to correlate with functional differences. For example, when human ASC are maintained in 3D spheroids *in vitro*, their adipokine secretion is an order of magnitude greater than an equivalent number of ASC in 2D cultures [6].

Increased attention to “organ on a chip” technologies has promoted advances in 3D culture approaches for adipose tissue and cells [7–10]. These approaches combine murine cell lines (3T3-L1) or primary murine or human ASC with biomaterial scaffolds derived from hyaluronic acid or silk to create *in vitro* or *in vivo* microphysiological systems that mimic either white or beige/brown adipose tissue [7–10]. In a recent report, Lau et al. described a method for maintenance of human adipose tissue as “SWAT” or “sandwiched white adipose tissue” cultures [11]. Intact fragments of adipose tissue were maintained for up to 8 weeks *in vitro* as organoid cultures between sheets of primary cultured human ASC [11]. This method has a value for pathophysiological and pharmacological investigation of primary human adipose tissue. Nevertheless, it does require direct collaboration with a research-oriented plastic or general surgeon and an approved Institutional Review Board protocol. For laboratories located on campuses without a medical school or research hospital, these restrictions may make the SWAT approach impractical. Therefore, the current study was undertaken to provide an alternative 3D human adipose model for this segment of the research community.

This manuscript describes a method employing cryopreserved primary human stromal vascular fraction (SVF) cells and a human blood product-derived biological scaffold to create a xenoprotein-free 3D adipose depot *in vitro* that is suitable for investigating human adipose biology. The construct is established using cryopreserved human SVF cells which contain heterogeneous subpopulations of viable cells that are representative of individual donor demographics. The resulting adipose constructs self-assemble into spheroids within 1 week of culture without the need for laborious or expensive protocols and have been validated relative to 2D cultures based on flow cytometry, confocal and cryogenic electron microscopy/immunofluorescence, in vivo behavior, and functional assays (adipokine secretion, glucose uptake, and lipolysis).

## 2. Materials and Methods

### 2.1. General

Unless otherwise noted, all materials were obtained from Thermo Fisher Scientific and its subsidiary companies or from LaCell LLC. Specimens of human lipoaspirate were donated by healthy individuals undergoing elective liposuction with written informed consent under a protocol reviewed and approved by the Western Institutional Review Board (WIRB, Puyallup WA) (IRB Tracking # 20130449).

### 2.2. Stromal Vascular Fraction (SVF) Cell Isolation

Human SVF cells were isolated from lipoaspirate specimens of subcutaneous adipose tissue by enzymatic digestion using collagenase type 1 (Worthington Biochemical, Lakewood, NJ) according to published protocols [12, 13]. The resulting SVF cell viability and quantification were determined using Live/Dead Solution assay (LaCell Catalog # LaLD-2) and fluorescent microscopy hemocytometer inspection.

### 2.3. Cell Proliferation Assay

Immediately after thawing, metabolically active SVF cells were quantified with using an ethidium bromide/acridine orange-based Live/Dead Solution (LaCell Catalog # LaLD-2) viability staining protocol. Cells were then plated in triplicate at a concentration of 64,000 cells/well in a 96-well culture plate with 1 mL of culture medium supplemented with the corresponding amount of ObaGel™ (LaCell Catalog # LaObG-10) (0% for cultures and silk scaffolds, 7.5% for gel). Cells were cultured for 3, 5, and 7 days. After culture, cells were incubated with 20 *μ*L Cell Titer Blue working reagent which was added to wells for four hours. Fluorescence was quantified using an excitation wavelength of 560 nm and an emission wavelength of 590 nm. Control scaffolds and wells were analyzed similarly to adjust for background fluorescence.

### 2.4. Colony-Forming Unit Fibroblast (CFU-F)

Immediately after thawing, metabolically active cells were quantified using an ethidium bromide/acridine orange Live/Dead Solution (LaCell Catalog # LaLD-2) viability staining protocol. Cells were then plated in 100 mm culture dishes at a concentration of 50 or 100 cells/plate in LaCell Stromal Medium (LaCell Catalog # LaSM-500) and fed every 3 days. After 12-14 days, cultures were fixed in formalin and stained using a Toluidine Blue Staining Solution (LaCell Catalog # LaTB-50) protocol.

### 2.5. Adipogenic Differentiation

For 2-dimensional cultures, immediately after thawing, cells were seeded into 24-well plates at a concentration of 3 × 10^4^ cells/cm^2^ for 24 hours to allow for cell attachment. After the overnight incubation, medium was removed, replaced with fresh AdipoQual™ Medium (LaCell Catalog # LaADM-500), and fed with the same medium every 3 days for up to two weeks.

### 2.6. ObaGel 3D Static Adipogenic Cultures

**Figure 1:**
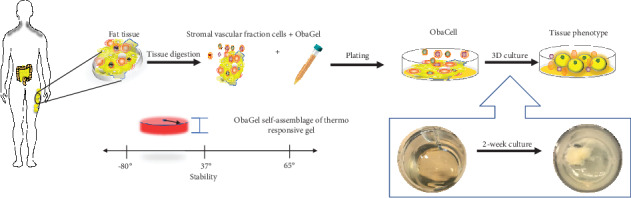
Summary figure representing the overall process for developing static fat-on-a-chip cultures using ObaGel.

For 3-dimensional cultures using ObaGel, a summary figure has been provided to represent the overall process for developing static fat-on-a-chip cultures (see ObaGel Figure 1). ObaGel is a thermoresponsive gel that self-assembles into a complex, self-assembling tissue matrix with a structure that is driven by cell-mediated extracellular matrix remodeling. Briefly, ObaGel was thawed overnight on ice (4°C) and diluted with StromaQual (LaCell Catalog # LaSM-500) media to desired concentrations (0% to 25% (*v*/*v*)). SVF cells were isolated from digested subcutaneous adipose tissue according to published protocols and as described in Stromal Vascular Fraction (SVF) Cell Isolation above. Cells were mixed with the ObaGel/medium mixture at desired concentrations and immediately seeded into 24-well multidishes in 1 mL volumes, or as indicated for each study. The cell suspension was pipetted directly into the wells according to the desired volume per well. Matrix polymerization occurred immediately after transfer to 37°C cell culture incubators. Cells/ObaGel mixtures, referred to as ObaCell™, were cultured for two weeks to allow cell-mediated matrix remodeling, robust growth, and priming for adipose tissue functionality. Constructs were then induced along the adipogenic lineage with exposure to AdipoQual (LaCell Catalog # LaADM-500) medium supplemented with ObaGel at the same indicated concentrations per study (see *Cell Proliferation Assay,, Functional Analytical Assays (Glucose Uptake, Lipolysis, and Adipokine Secretion), and Confocal and Electron Microscopy).*

### 2.7. Oil Red O Staining

Intracytoplasmic lipid deposition was assessed using an Oil Red O Staining Solution (LaCell Catalog # LaORO-50) protocol: Cells cultured for 3 days in 24-well plates were first rinsed with PBS thrice and then fixed for 15 minutes with 10% (*v*/*v*) formalin solution. Monolayers were then washed three times with PBS to remove formalin solution. Fixed monolayers were then incubated for 45 minutes at room temperature with the ORO Staining Solution. After 45 minutes, excess staining solution was aspirated and monolayers were rinsed four times with PBS to remove any remaining staining solution. The ORO-stained monolayers were visualized using phase contrast microscopy (Motic AE2000, Richmond, British Columbia, Canada).

### 2.8. BODIPY Staining

For ObaCell cultures, accumulated lipid was also visualized using BODIPY lipophilic fluorescent staining. ObaCell cultures were fixed in 10% buffered neutral formalin overnight at 4° Celsius and rinsed with distilled water. BODIPY working solution was added to cultures and incubated for an additional 24 hours. Cultures were rinsed with distilled water and counterstained with Hoechst nuclear dye according to the manufacturer's protocol. Images were taken using confocal laser microscopy.

### 2.9. Functional Analytical Assays (Glucose Uptake, Lipolysis, and Adipokine Secretion)

ObaCell cultures were established according to Methods in *Cell Culture in 2- and 3-Dimensional Configurations.* 100 *μ*L of the conditioned medium from ObaCell cultures was collected at days 7, 14, 21, and 28 (*N* = 6 replicates per sample condition) and transferred to 96-well dishes for assays. Protocols were followed as specified by manufacturers for measuring glucose uptake, lipolysis, adiponectin, or leptin secretion.

### 2.10. Flow Cytometry: 2D SVF Cultures

Single-cell suspensions were prepared according to a standard protocol for 2D cultures and ObaGel 3D static cultures. For 2D cultures, 10^6^ SVF cells were plated in T-75 flasks and cultured in control or differentiation medium. The SVF were then resuspended using a standard trypsin-EDTA protocol, washed with prechilled phosphate-buffered saline (PBS) supplemented with 1% bovine serum albumin (BSA), distributed into separate Eppendorf tubes, and incubated with an antibody in darkness for 30 minutes. Following the incubation periods, cells were washed with PBS three times and fixed using 10% formalin. Samples were analyzed using a FACSCalibur cytometer (Becton Dickinson Immunocytometry Systems). The following markers were analyzed: CD29+/CD105+/CD45-/CD34+/CD73+/CD90+.

### 2.11. Flow Cytometry: ObaGel Cultures

ObaGel cultures were established using the methods described in ObaGel 3D Static Adipogenic Cultures above. Cells were cultured and harvested at the time points indicated per study by adding an Obazyme digestion agent (Obatala Sciences Catalog # ObaZYM-15) directly to the wells in a 1 : 1 ratio with ObaCell static culture volumes for 15 minutes for one-day cultures and up to one hour for two-week established cultures. The resulting single-cell suspensions were collected, centrifuged at 300 x g (1200 rpm) for 5 minutes, and resuspended in PBS supplemented with 1% BSA. Cells were then distributed into separate Eppendorf tubes and incubated with the same antibodies and protocol indicated above in Flow Cytometry: 2D SVF Cultures.

### 2.12. Confocal and Electron Microscopy

Confocal microscopy was performed on ObaGel- and silk-based *in vitro* adipogenic differentiated constructs (see Adipogenic Differentiation) and explants following 6-week *in vivo* implantation and staining with BODIPY lipophilic dye. Confocal research was conducted using a Nikon A1 LASER Ti-E microscope system. The tissue was imaged in Nunc 35 mm glass bottom dishes, at 10x and 20x oil-free objectives, using the FITC and DAPI LASERs. Cryoscanning electron microscopy was conducted on ObaGel constructs and silk scaffolds that were seeded with human SVF cells following a 2-week adipogenic differentiation. Cryo-SEM structure research was done with Gatan 2500 alto Cryo-system and Hitachi S-4800 SEM. The tissue was cut to 7 × 5 × 5 (mm) and laid about 5 mm high above a cryogenic SEM sample holder surface, clamped and glued into the holder, and then frozen in slushed liquid nitrogen at approximately -210°C for about 30 seconds. The frozen tissue was transferred in vacuum to the prechamber attached to the SEM and then fractured and sublimed for 5 minutes at -95°C. After coating for 88 seconds at -130°C with Pt/Pd, the sample was moved to the SEM and observed at 3 kV at -130°C.

### 2.13. Cell Profiler Image Analysis

Image analysis was performed using CellProfiler v2.2.0. Raw images from all image sets were imported into CellProfiler and then separated into discrete DAPI and FITC channels. For each channel, the total image intensity was measured using the MeasureImageIntensity module. Individual image sets were then run through a series of modules to identify the objects in them.

For the 2D versus 3D culture image set, the DAPI channel was run through the IdentifyPrimaryObjects module using a global threshold strategy and an Otsu three-class thresholding method with pixels from the middle intensity assigned to the background. Intensity was used to distinguish individual objects after thresholding. The FITC channel from the same set was run through the IdentifyPrimaryObjects module with the same settings, except that a Laplacian of Gaussian method was used to distinguish between objects after thresholding and the resulting shapes were used to draw dividing lines.

For the white three-dimensional culture image set, the DAPI and FITC channels were subtracted from each other using the ImageMath module to improve contrast. Both the DAPI minus FITC and the unaltered DAPI channels from this set were then run through the IdentifyPrimaryObjects module using an adaptive threshold strategy and an Otsu three-class thresholding method with pixels from the middle intensity assigned to the background. The FITC minus DAPI image set and the unaltered FITC channels from the white set were run through the IdentifyPrimaryObjects module using a global threshold strategy and an Otsu three-class thresholding method with pixels from the middle intensity assigned to the background. The unaltered DAPI and FITC channels were then averaged using the ImageMath module. The resulting images were then run through the IdentifyPrimaryObjects module using an adaptive threshold strategy and an Otsu three-class thresholding method with pixels from the middle intensity assigned to the foreground. All three sets of modules used for the white image set used intensity to distinguish individual objects after thresholding.

For the silk- versus ObaGel-based fat formation image set, the DAPI channel was run through the IdentifyPrimaryObjects module using an adaptive robust background thresholding strategy. For this set, intensity was used to identify objects after thresholding and their shape was used to draw dividing lines. The FITC channel from this set was run using a global threshold strategy and an Otsu three-class thresholding method with pixels from the middle intensity assigned to the background. For this set, intensity was used to distinguish individual objects after thresholding.

The image area occupied by identified objects in all image sets was measured using the MeasureImageAreaOccupied modules. Objects touching the border of the image from all images were then discarded, and the remaining objects were quantified with the MeasureObjectIntensity and MeasureObjectSizeShape modules. Images were saved after splitting the channels and each object identification module. Results from all modules were exported to Excel.

### 2.14. In Vivo 3-Dimensional Construct Implants

Implants were performed using ObaGel or hexafluorisopropanol (HFIP) silk scaffolds (Tufts University Tissue Engineering Research Center, Medford, MA) seeded with or without SVF cells in 6-8-week-old C57BL/6 female mice (Charles River Laboratories, Wilmington, MA) in accordance with a Tulane University Institutional Animal Care and Use Committee reviewed and approved protocol (#4302R; March 16, 2016) as previously described [14]. Studies were conducted in the Tulane University vivarium with veterinary care provided daily by the Department of Comparative Medicine.

### 2.15. Statistical Analyses

Values are presented as the mean ± standard deviation (SD) unless otherwise noted. Statistical significance was determined based on Student *t*-test and 1-way or 2-way ANOVAs with multiple comparisons tests performed. For multiple comparisons using statistical hypothesis testing, Sidak post tests were used with multiplicity-adjusted *p* values reported, for *p* values < 0.05. Calculations were performed using Prism GraphPad software (La Jolla California, USA).

## 3. Results

### 3.1. Immunophenotype of Human SVF Cells

**Figure 2:**
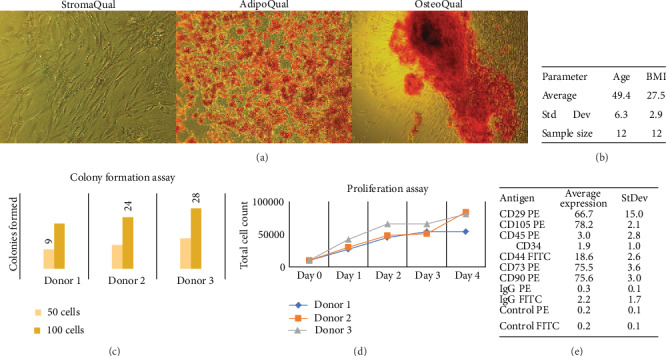
Demographic characteristics of donors and SVF cells utilized in the study. Human SVF cells were characterized on the basis of (a) adipogenic and osteogenic differentiation, (b) donor demographics, (c) colony formation, (d) proliferation, and (e) cell immunophenotype. Donor demographics are as follows (in mean ± SD): age (49.4 ± 6.3), body mass index (BMI 27.5 ± 2.9).

Freshly isolated and cryopreserved human SVF cells (*N* = 12 donors) utilized in the study were characterized on the basis of adipogenic and osteogenic differentiation, colony-forming unit fibroblastic (CFU-F) formation, proliferation, and immunophenotype at passage 0 (P0) of the culture (Figure 2). Our labs have worked extensively with SVF cells and developed standard operating procedures to characterize the subpopulations that exist within this heterogeneous fraction (Frazier et al. 2018). The donors had a mean age (± SD) of 49.4 ± 6.3 years and a mean body mass index (± SD) of 27.5 ± 2.9 kg/m^2^. Donor immunophenotypes consisted of hematopoietic/stromal (CD90: 75.6% ± 3.0%), cell-cell and cell-surface adhesion (CD29: 66.7% ± 15.0%; CD44: 18.6% ± 2.6%), leukocyte common antigen (CLA; CD45: 3.0% ± 2.8%), and stem/progenitor (CD34: 1.9% ± 1.0%; CD73: 75.5% ± 3.6%, and CD105: 78.2% ± 2.1%) markers immediately postthaw.

### 3.2. Polymerization of ObaGel as a Thermoresponsive Gel Is a Cell-Driven Process

**Figure 3:**
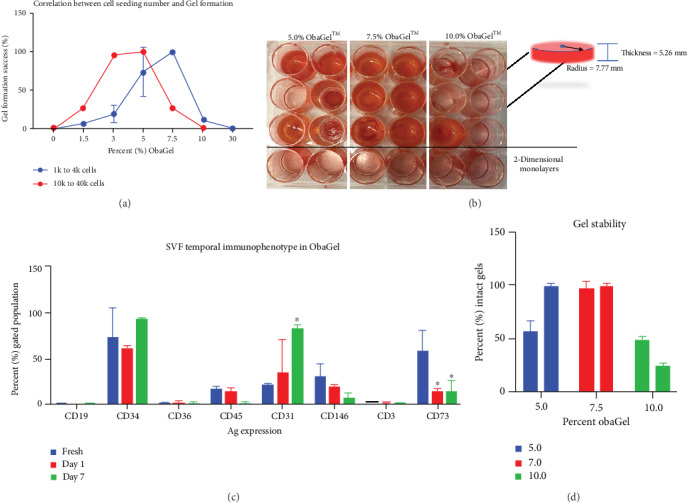
Temporal immunophenotype and gelling behavior of SVF cells cultured in 3 dimensions in human-derived ObaGel. Studies investigating the correlation of cell seeding density and ObaGel concentration to gel stability were performed (a), and whole plate images were taken (b). Gel stability was measured and reported as a percentage (%) total gelling. Investigations into impact on SVF immunophenotype from freshly isolated SVF cells compared to ObaGel cultures at days 1 and 7 (c). 100% gel formation was maintained in 7.5% ObaGel-supplemented medium (*v*/*v*), in the absence of any additional supplementary growth factors (d). Values reported as mean ± SD. ^∗^*p* < 0.05.

Studies investigating the correlation of cell seeding density and ObaGel concentration to gel stability were performed (Figure 3(a)). ObaGel cultures were seeded in increased concentrations, ranging from 500 to 20 k cells per cm^2^ of 24-well multiwells. Gel stability was measured by observing the number of wells with gels maintained at a consistent volume postseeding, divided by the total number of seeded wells, and reported as a percentage (%) total gelling. 100% gel formation was maintained in 7.5% ObaGel-supplemented medium (*v*/*v*), in the absence of any additional supplementary growth factors (Figures 3(a) and 3(d)).

### 3.3. Immunophenotype of ObaGel-Cultured Human SVF Cells

Flow-based immunophenotyping of SVF cells cultured in 7.5% ObaGel determined that the cells remained viable and continued to display heterogeneity (Figure 3(c)). Compared to the immunophenotype of freshly thawed cells, the expressions of antigens associated with myeloid (CD19), stem/progenitor (CD34), preadipocyte (CD36), hematopoietic (CLA; CD45), pericytic (CD146), and lymphoid (CD3) identity were not significantly different after cryopreservation, thawing, and culturing in ObaGel for one-week postseeding. In contrast, SVF cells significantly upregulated the expression of CD31, a marker of endothelial progenitors (freshly thawed SVF: 23.5% ± 3.4% vs. day 7 ObaGel culture: 87.0% ± 7.9%), and significantly downregulated the expression of CD73, a marker of lymphocytic differentiation (freshly thawed SVF: 54.5% ± 12.2% vs. day 7 ObaGel culture: 25.78% ± 9.2%).

### 3.4. 3D Culture in ObaGel Stimulates Robust Human SVF Cell Proliferation and Self-Assembly into Spheroids

**Figure 4:**
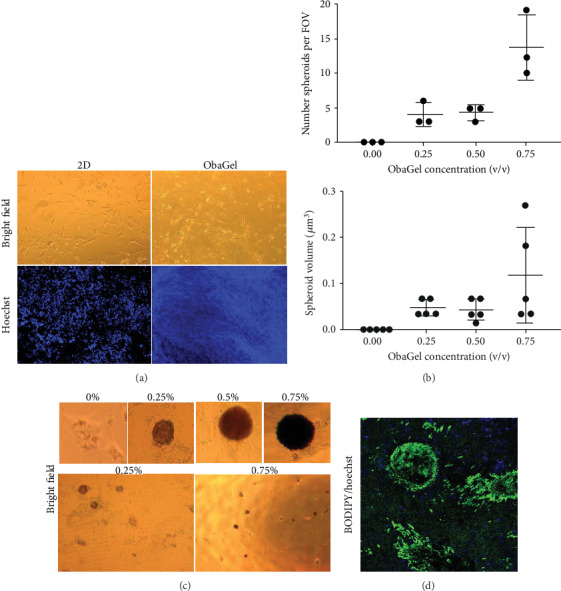
Comparison of SVF proliferation, spheroid volume and formation, and adipogenic functionality in 2D culture and ObaGel. (a) Bright field and confocal microscopy images of SVF cell proliferation in 2D culture and ObaGel. Cells were seeded in traditional 2D media or ObaGel, cultured for 5 days, and stained with the Hoechst dye. Robust cell proliferation occurs in ObaGel compared to 2D culture, as indicated by Hoechst positivity. (b, c) report photomicrographs and respective quantification that reflect a positive correlation between ObaGel concentration and SVF spheroid size, density, and volume within 5-7 days of culture. (d) demonstrates that adipogenesis occurs within spheroids as early as day 3 of differentiation. Values reported as mean ± SD. ^∗^*p* < 0.05 when compared to the freshly thawed cells or silk constructs.

Cellular proliferation was substantially higher in ObaGel compared to traditional 2D culture in medium supplemented with fetal bovine serum (Figure 4(a)). Further investigation into the impact of ObaGel on SVF cell behavior revealed a concentration-dependent development of spheroids upon exposure to lower concentrations of ObaGel, as early as 5 to 7 days of culture (Figure 4(c)). Spheroid size and volume were increased as a function of ObaGel concentration between percentages of 0.25% and 0.75%. Adherent cell populations decreased with increasing ObaGel concentrations, reflecting an indirect relationship existing between cell attachment and ObaGel concentration (data not shown). Adipogenic differentiation of the ObaGel cultures revealed an ability to induce a robust intracytoplasmic lipid accumulation within the adipose stromal/stem populations in spheroids at low ObaGel concentrations in cultures (Figure 4(d)).

### 3.5. Compared to 2D Culture and Silk Scaffolds, ObaGel Supports Robust Lipid Accumulation, More Complex Network Formation, and Maintenance of a Cell Surface Antigen Profile Similar to Freshly Isolated SVF Cells

**Figure 5:**
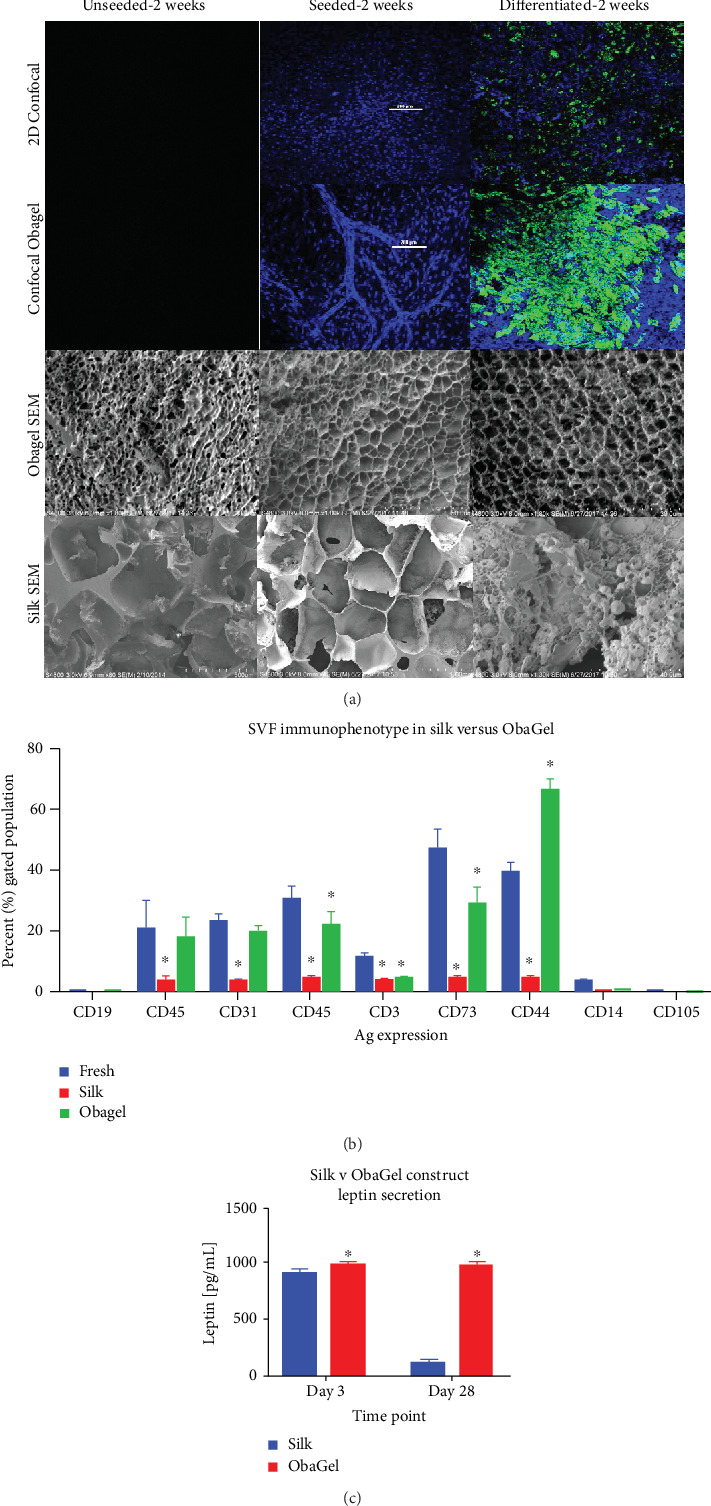
A comparison of the *in vitro* behavior of human SVF cells when cultured in 2-dimensional conditions and in 3 dimensions when seeded on either silk scaffolds or cultured in ObaGel for 2 weeks. (a) Confocal and cryoscanning electron microscopy (cryo-SEM) images of SVF cell seeding, proliferation, and adipogenic differentiation behavior in traditional 2D culture, silk scaffolds, and ObaGel. (b) SVF immunophenotype following 1 week of culture reveals that ObaGel is superior in maintaining a heterogenic immunophenotype similar to freshly isolated cells and supports the expansion of CD44-positive subpopulations (b), and adipokine functionality (c) reports the measure of leptin secretion from adipogenic differentiated cells seeded on silk or with ObaGel are early (day 3) and late (day 28) time points of differentiation. Immunophenotype values reported as mean (*μ*) ± standard deviation (SD). ^∗^*p* < 0.05.

Previous 3D adipose studies conducted by our lab and our collaborators employed hexafluoro-isopropyl- (HFIP-) treated silk porous scaffolds to support the growth and adipogenic differentiation of SVF cells for an *in vitro* adipose tissue model [14–16]. The present study extended these observations by comparing the behavior of human SVF cells when cultured in 2-dimensional conditions and in 3-dimensions when seeded on either silk scaffolds or cultured in ObaGel for 2 weeks. SVF cell immunophenotype, adipogenic capacity, and adipokine functionality were investigated (Figure 5). At two weeks postseeding, undifferentiated SVF cells demonstrated robust differences in proliferation, similar to observations in Figure 3. Cell-mediated matrix remodeling was supported in ObaGel cultures, where complex networks were formed as early as 8-10 days (Figure 5(a), ObaGel confocal image). Formed networks increased their complexity and size by 14 days of culture (Figure 5(a)). This phenomenon was also noted to be dependent on ObaGel concentration, initially appearing at concentrations as low as 1.5% (*v*/*v*; data not shown). Likewise, intracytoplasmic lipid accumulation was significantly higher in ObaGel cultures compared to 2D cultures (Figure 5(a)). Lipid droplets were observed near the top of ObaGel cultures and continuing to accumulate in number, starting from day 5.

Similar to previous characterization studies (Figure 3), ObaGel did not significantly impact SVF heterogeneity. Compared to the immunophenotype of freshly thawed cells, the expression of antigens associated with all of the lineages immunoassayed was significantly decreased in silk after 2 weeks of culture (Figure 5(b)). In comparison, ObaGel a higher degree of SVF heterogeneity, with a significant increase in the expression of the stromal marker CD44 and decreases in the expressions of pericytic (CD146), lymphoid (CD3), and lymphoid differentiation (CD73) markers (Figure 5(b)). Similar to previous reports, adipokine secretion was supported during early adipogenesis in silk. However, ObaGel cultures secreted higher levels of leptin than silk 3D cultures at both early (day 3: silk, 923.4 pg/mL ± 29.0 pg/mL; ObaGel, 997.5 pg/mL ± 21.6 pg/mL) and late (day 28: silk, 118.8 pg/mL ± 17.2 pg/mL; ObaGel, 981.8 pg/mL ± 25.2 pg/mL) time points.

### 3.6. ObaGel Cultures Exhibit Adipose Functionality Relative to Physiologically Relevant Adipose Explants and In Vivo Serum Levels

**Figure 6:**
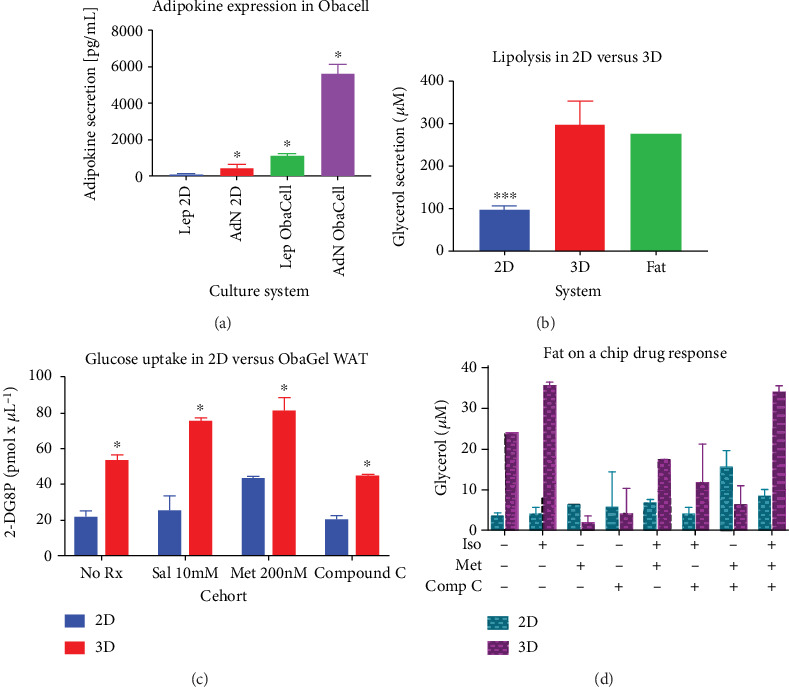
ObaGel cultures exhibit physiologically relevant functionality. Adiponectin (AdN) and leptin (Lep) secretions were measured from traditional cell cultures (2D) compared to three-dimensional adipose cultures (ObaCell) after 21 days of culture. Values reported are in pg/mL conditioned medium and are reported as mean (*μ*) ± standard deviation (SD). ^∗^*p* < 0.05. Adipose tissue functionality demonstrated with measurement of glycerol secretion in a conditioned medium from cells differentiated in two dimensions (2D) compared to ObaGel after 14 days of culture. ObaGel cultures demonstrated higher sensitivity to AMPK-targeting compounds isoproterenol, metformin, compound C, and IBMX. Values reported are in pg/mL conditioned medium and are reported as mean (*μ*) ± standard deviation (SD). ^∗^*p* < 0.05.

Adiponectin (AdN) and leptin (Lep) secretions were measured from 2D cell cultures compared to ObaGel cultures after 21 days of culture. ObaGel cultures consistently secreted higher adipokine levels compared to 2D cultures, both in the case of AdN (2D: 420.3 pg/mL ± 199 pg/mL; ObaGel: 5630 pg/mL ± 504.9 pg/mL; Figure 6(a)) and Lep (2D: 84.1 pg/mL ± 39.8 pg/mL; ObaGel: 1126 pg/mL ± 101.1 pg/mL; Figure 6(a)). AdN serum concentrations in humans are reported in the literature as a median of 41.47 ng/mL (41,470 pg/mL), with a range of 6.01 ng/mL-145.40 ng/mL (6,010 pg/mL–145,400 pg/mL) [17]. Lep serum concentrations in humans are reported in the literature as a median of 221.85 pg/mL, with a range of 45.68 pg/mL–894.5 pg/mL [17]. ObaGel culture functionality was further investigated with measurement of glycerol secretion in the conditioned medium after 14 days of culture. Free glycerol levels were substantially higher in ObaGel cultures compared to 2D cultures (2D: 96.7 *μ*M ± 10.0 *μ*M; ObaGel: 298.5 *μ*M ± 54.8 *μ*M; Figure 5(b)). In comparison, detected glycerol levels from excised murine adipose was averaged at 276 *μ*M.

### 3.7. ObaGel Cultures Exhibit Increased Sensitivity to AMPK-Targeting Compounds Prescribed for Regulating Insulin Sensitivity

In the absence of any compounds, ObaGel 3D cultures demonstrated higher functionality as measured by 2-deoxy-D-glucose uptake activity (2D: 22.1 pM/*μ*L ± 3.1 pM/*μ*L; ObaGel: 53.7 pM/*μ*L ± 2.8 pM/*μ*L; Figure 6(c)). Further studies evaluated ObaGel cultures treated with AMPK-activating compounds isoproterenol and metformin, and an AMPK inhibitor, compound C (Dorsomorphin). Exposure to the compounds revealed increased sensitivity in ObaGel cultures compared to 2D (Figure 6(c)), at doses reported to correlate with pharmacologically relevant levels found in the literature. Compound C significantly attenuated 2-DG6P detection by 9% compared to baseline and by 36% compared to metformin-mediated glucose uptake (Figure 6(c)). Likewise, ObaGel cultures demonstrated significantly higher glycerol secretion at both baseline levels (2D: 3.4 *μ*M ± 0.8 *μ*M vs. ObaGel: 24 *μ*M ± 0.7 *μ*M; Figure 6(d)), and when stimulated by isoproterenol (2D: 4 *μ*M ± 1.6 *μ*M vs. ObaGel, 35.2 *μ*M ± 0.8 *μ*M; Figure 6(d)).

### 3.8. ObaGel Demonstrates Robust Vascular Potential In Vivo

**Figure 7:**
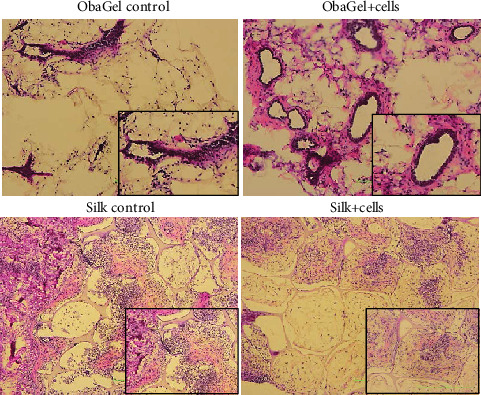
Implantation of ObaGel for the development of a humanized fat pad in mice. Scaffolds remained in mice for 6 weeks. Hematoxylin and eosin staining revealed a dense confinement of proliferating cells within the silk scaffolds. In contrast, ObaGel implants contained large blood vessels that were clearly integrated functionally within larger, more mature adipocytes.

We investigated the ability of human SVF cells to form functional fat in nude mice when seeded in ObaGel as compared to silk sponge scaffolds (Figure 7). Negative controls included injections of ObaGel alone or empty silk scaffolds. Scaffolds remained in mice for 6 weeks, similar to previously published silk adipose construct studies [14]. Hematoxylin and eosin staining revealed a dense confinement of proliferating cells within the silk scaffolds. In contrast, ObaGel implants contained larger structures that were clearly integrated functionally within larger, more mature adipocytes (Figure 7).

### 3.9. In Vivo Analysis of Implants of Human SVF Cell ObaGel Constructs in Immunodeficient Mice

**Figure 8:**
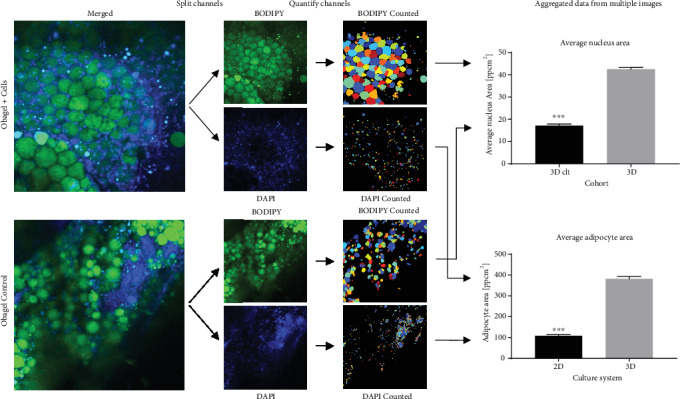
In vivo analysis of implants of human SVF cell ObaGel constructs in immunodeficient mice. BODIPY lipophilic staining was performed in explants removed from nude mice following 6-week implantation. Images were taken by confocal laser microscopy and quantified using CellProfiler v2.2.0 software. Values reported are in pg/mL conditioned medium and are reported as mean (*μ*) ± standard deviation (SD). ^∗^*p* < 0.05.

BODIPY lipophilic staining was performed in explants removed from nude mice following 6-week implantation. Images were taken by confocal laser microscopy and quantified using CellProfiler v2.2.0 software. Control implants were ObaGel implants injected in the absence of cells. Quantified results demonstrate the presence of significantly larger adipocytes that contained larger nuclear areas within ObaGel/SVF explants compared to ObaGel control explants (Figure 8).

## 4. Discussion

International regulatory agencies have begun to advocate for the increased incorporation of 3D human cell-based models as a central screening assay within the pharmaceutical drug discovery process [18]. It is anticipated that such 3D models will reduce the reliance on *in vivo* animal models while increasing the efficiency for the identification of effective therapeutic target compounds [19]. As a result, investigators have begun to combine natural or synthetic scaffolds with primary human tissue-derived cells to create novel 3D organoids. The current manuscript demonstrates the utility and functionality of a 3D construct containing both primary human adipose-derived cells and a natural scaffold as an *in vitro* model for adipose tissue depots (Figure 2). Based on morphological criteria, the ObaGel scaffold was superior to a silk scaffold in supporting the adipogenic differentiation of human SVF cells. The ObaGel in the presence of SVF cells promoted the formation of vascular-like structures within the 3D constructs and gelled in a time- and concentration-dependent manner. Throughout a one-week period, adipose-derived SVF cells within the ObaGel constructs continued to express surface antigens associated with stem cell (CD34), endothelial cell (CD31), and ASC (CD73) phenotypes. Relative to 2D cultures, SVF cells in 3D ObaGel constructs displayed increased adipogenesis based on both number and lipid vacuole size, consistent with the differentiation of mature “signet ring” adipocytes. Likewise, the 3D ObaGel constructs were superior to 2D cultures with respect to glucose uptake and adipokine (leptin) secretion as well as lipolytic response to agents such as isoproterenol, metformin, and compound C. In the presence of selective inductive cocktails, the SVF cells in 3D ObaGel constructs expressed biomarkers consistent with white adipose tissue (WAT) depots. Together, this data documents the efficacy and utility of reagents and methods for the reproducible construction of 3D scaffolds displaying features of human adipose depots suitable for pharmaceutical and metabolic *in vitro* studies.

Prior studies have reported protocols to culture 3D human adipose depots *in vitro*. Among the most well-established models has been the maintenance of intact adipose tissue fragments by a variety of culture methods as described by Fried, Lau, Miyazaki, and others [11, 20–22]. In these methods, adipose tissue is minced into cubes of 1-2 mm in any dimension and incubated as suspensions forced below the surface of the culture medium by a screen, as explant “ceiling cultures” within flasks completely filled with medium, in the presence of continuous agitation, or sandwiched between layers of culture expanded ASC. These models rely on the investigator's ready access to freshly isolated adipose tissue specimens from donors of appropriate demographic characteristics (BMI, age, gender, ethnicity, and disease status). While a number of studies have demonstrated the feasibility of cryopreserving intact adipose tissue fragments for extended periods of time, only ~50% of the SVF cell population within these tissues remain viable [23]. This contrasts with cryopreservation of isolated SVF cells and ASC which retain postthaw viability levels of 80% or better, depending on the isolation process and cryopreservation agent [23–27]. It is noteworthy that in the current study, the SVF cell immunophenotype differed between freshly prepared and cryopreserved/thawed cells. While there is clear utility for the cryopreserved SVF cells and tissues, each may face some limitations.

Likewise, multiple independent investigators have employed spheroids as an *in vitro* 3D culture model [6, 28–31]. While spheroids could be prepared using hanging drop methods and low adhesion tissue culture surfaces, they could also be obtained using polymer microparticles [6, 28, 30, 31]. These studies further demonstrated that 3D spheroids secreted significantly greater quantities of adipokines [28] and wound healing-associated proteins [6] relative to 2D cultures containing comparable total cell numbers. Additionally, the adipose-derived cells in the 3D spheroids displayed superior brown adipogenesis relative to 2D culture models [28]. Alternative 3D culture methods, including bioprinting, have likewise displayed superior brown adipogenesis relative to 2D systems *in vitro [*32*–*34*]*. Several natural and synthetic biomaterials have been used here to support 3D adipogenic constructs *in vitro*. A growing body of literature has evaluated decellularized adipose tissue extracellular matrix as a biomaterial scaffold to promote 3D models for both brown and white adipogenesis [35–43]. Likewise, 3D silk scaffolds in combination with adipose-derived cells have been demonstrated to support adipogenesis [7, 14–16, 44–47].

While the current study validates the utility of ObaGel and SVF cells as an *in vitro* human adipose depot model, further studies are needed. In addition to the current work's examination of the 3D model's response to metformin and isoproterenol, the system needs to be tested more thoroughly with a wider panel of drugs with established pharmacokinetic and pharmacodynamic profiles in adipose tissue. While the system has been established firmly using SVF cells isolated from subcutaneous adipose depots, similar analyses should be performed using SVF cells isolated from adipose depots in the viscera, omentum, and around vital organs. Likewise, studies need to be performed evaluating the differentiation of human SVF cells in ObaGel along the beige/brown adipocyte lineage pathway. Moreover, since human ASC and SVF cells can be differentiated along the chondrogenic, endothelial, and osteogenic lineages, it also remains to be determined if the ObaGel/SVF cell scaffolds can be used to model the bone, cartilage, and/or vascular networks or depots *in vitro*. Finally, while the current model relies on a static culture system, its performance within a perfusion bioreactor is feasible and needs to be evaluated. These issues provide a potential framework for the design of future experiments employing the human SVF cell and ObaGel constructs as an “off the shelf” system with an extended shelf life that is superior to the more laborious and more expensive methods that rely heavily on superimposed cellular dynamics and plasticware with specialized surface coatings.

## 5. Conclusions

The current study demonstrates that a combination of primary SVF cells and ObaGel scaffolds can be used to create a 3D *in vitro* construct with functional properties mimicking a humanized adipose depot. The ready availability of a 3D model as opposed to a 2D adipocyte model offers investigators an alternative model that more closely approximates the *in vivo* white adipose tissue microenvironment. Further studies will be necessary to characterize the SVF cell/ObaGel model with respect to beige/brown adipose tissue differentiation.

## Data Availability

The data used to support the findings of this study are included within the article.
